# Associations of hemoglobin trajectories and time in target hemoglobin range with adverse pregnancy outcomes: a real-world pregnancy records-based study

**DOI:** 10.3389/fgwh.2025.1682957

**Published:** 2025-11-21

**Authors:** Pinggui Zhang, Yuting Shen, Gang Zou, Xinli Xiang

**Affiliations:** 1 Shanghai First Maternity and Infant Hospital Affiliated to Tongji University School of Medicine, Shanghai, China; 2 Department of Obstetrics and Gynecology, Shanghai Ninth People’s Hospital Affiliated to Shanghai Jiao Tong University School of Medicine, Shanghai, China

**Keywords:** hemoglobin trajectory, adverse pregnancy outcome, real world, time in target range, prenatal care

## Abstract

**Objective:**

This study aimed to evaluate associations of hemoglobin trajectories during pregnancy and time spent within clinically recommended hemoglobin ranges (Hb-TITR) with adverse pregnancy outcomes.

**Methods:**

A retrospective cohort study was conducted using pregnancy records from Shanghai First Maternity and Infant Hospital (from January 2022 to December 2024), involving 7,653 pregnant women with complete serial hemoglobin measurements. Hemoglobin trajectories were categorized as stable, descending, or ascending. Hb-TITR was calculated based on clinically recommended hemoglobin ranges throughout pregnancy. Logistic regression analyses were performed to determine associations with adverse outcomes, adjusting for maternal age, pre-pregnancy BMI, and gestational age.

**Results:**

Among participants, 85.2% exhibited stable hemoglobin trajectories, 10.4% descending, and 4.4% ascending. Descending trajectory was significantly associated with increased risk of composite adverse outcomes [adjusted odds ratio (OR) 3.92; 95% CI, 3.30–4.66] compared with the stable group. Conversely, ascending trajectory showed no significant risk elevation (OR 1.13; 95% CI, 0.83–1.51). Reduced Hb-TITR (<80%) significantly increased the odds of adverse outcomes (OR 1.35; 95% CI, 1.10–1.65), whereas longer Hb-TITR was protective. Descending trajectories notably increased risks of PROM, cesarean section, postpartum hemorrhage, macrosomia, SGA, and LGA infants.

**Conclusion:**

Descending hemoglobin trajectories and reduced time spent within recommended hemoglobin ranges during pregnancy were significantly associated with increased risks of adverse maternal and neonatal outcomes, highlighting the importance of monitoring hemoglobin dynamics throughout pregnancy.

## Introduction

Hemoglobin levels during pregnancy play a critical role in maternal and fetal health. Both anemia and elevated hemoglobin concentrations have been independently associated with increased risks of adverse pregnancy outcomes, including preterm birth, low birth weight, fetal growth restriction, and maternal morbidity and mortality (1, 2). The American College of Obstetricians and Gynecologists defines anemia in pregnancy as a hemoglobin level less than 11 g/dL (hematocrit <33%) in the first or third trimesters, or less than 10.5 g/dL (hematocrit <32%) in the second trimester (3). However, the clinical significance of maintaining hemoglobin within this range throughout pregnancy remain uncertain. Existing literature often evaluates hemoglobin at a single point or limited intervals, potentially overlooking dynamic changes and temporal variations across gestational stages.

Recent advancements emphasize the significance of analyzing hemoglobin as a longitudinal trajectory rather than isolated measurements (4, 5). Hemoglobin trajectories capture dynamic physiological adaptations, including hemodilution and erythropoiesis, thus providing a more comprehensive understanding of maternal and fetal risks. Additionally, quantifying the duration (time) spent within recommended hemoglobin targets may offer valuable insights into the management of maternal health and prevention of pregnancy complications.

To date, limited studies have comprehensively explored how hemoglobin trajectories and the duration within clinically recommended hemoglobin ranges relate to adverse pregnancy outcomes, particularly utilizing real-world clinical data. Therefore, this study aimed to investigate the associations between distinct hemoglobin trajectories and time spent in target hemoglobin ranges during pregnancy with various maternal and neonatal adverse outcomes.

## Methods

### Study design and data source

This was a retrospective cohort study conducted using real-world pregnancy records from one obstetric campus of Shanghai First Maternity and Infant Hospital Affiliated to Tongji University School of Medicine. Data collection spanned from January 1, 2022 to June 30, 2022, involving women who completed pregnancy during this period and attended routine prenatal care at the facility. The Institutional Review Board (IRB) of the participating clinic approved the study protocol (KS21304, approved in 2021), and the requirement for informed consent was waived due to the retrospective nature and anonymization of data.

### Study population

**Figure 1:**
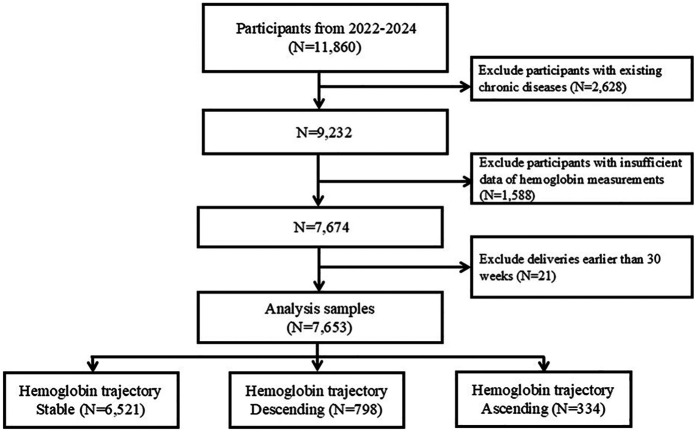
Study flow chart.

Women aged between 20 and above with complete pregnancy records, including complete serial hemoglobin measurements, were included. In total we have identified 11,860 pregnancy records from our electronic medical records (EMRs). Individuals who had existing chronic diseases prior to pregnancy (*n* = 2,628) or insufficient hemoglobin measurement data (*n* = 1,588) were subsequently excluded. Additionally, participants delivering 30 weeks before gestation (*n* = 21) were also excluded. After applying these criteria, a total of 7,653 participants remained and were included in the final analysis (Figure 1).

### Hemoglobin trajectory and time in target range (TITR) definitions

Hemoglobin collected from blood routine throughout pregnancy were collected at multiple routine prenatal and postpartum visits at first trimester, second trimester, third trimester, before delivery and right after delivery. Trajectories of hemoglobin were classified based on these 5 hemoglobin measurements.

Time in target hemoglobin range (Hb-TITR) was calculated as the percentage of pregnancy duration spent within the clinically recommended hemoglobin ranges as first trimester <110 g/L, second trimester <105 g/L and third trimester <110 g/L, of which the units were converted as routines in the practice in China. Participants were categorized based on Hb-TITR percentages into three groups: 100%, >80% to <100%, and <80% to provide clinically interpretable strata, in addition to analyses modeling Hb-TITR continuously. The reason why we used the corresponding cut points of Hb-TITR was because a large proportion of participants had a hemoglobin TITR of 100%. Therefore, these participants were considered as one group and the remaining participants were divided by the mean Hb-TITR among the population which is 80%.

Hb-TITR was thus calculated by a linear interpolation using the methods reported by Zhang et al. (6) Specifically, Hb-TITR was determined by calculating the number of days one individual was in the ranges at each prenatal visit (first trimester, second trimester, third trimester, and before delivery) and right after delivery after linear interpolation. We then calculated Hb-TITR as a percentage of time.

### Outcome measures

The primary outcome of interest was a composite of adverse pregnancy outcomes including gestational diabetes mellitus (GDM), hypertension, intrahepatic cholestasis of pregnancy (ICP), pulmonary embolism (PE), preterm rupture of membranes (PROM), cesarean section (C-section), small for gestational age (SGA), large for gestational age (LGA), preterm birth, preeclampsia, macrosomia, and postpartum hemorrhage (PPH) (7, 8). Preeclampsia was diagnosed as systolic blood pressure ≥140 mmHg and/or diastolic blood pressure ≥90 mmHg on two or more occasions with at least 6 h apart and proteinuria ≥1+ on dipstick or ≥300 mg on 24-hour urine collection. Preterm delivery was defined based on delivery earlier than 37 weeks of pregnancy. LGA was defined as birth weight above the 90th percentile for gestational age. SGA was defined as birth weight below the 10th percentile for their gestational age. Macrosomia was defined as a birth weight of 4,000 g. Excessive postpartum hemorrhage was defined as hemorrhage over 400 mL. PROM was defined as the rupture of the amniotic sac before the onset of labor.

### Covariates

Covariates included maternal age, pre-pregnancy body mass index (BMI), and gestational age at delivery. Maternal age was categorized as <30 and ≥30 years. BMI was categorized according to Chinese-specific criteria into <24 and ≥24 kg/m^2^ groups (9). GDM was diagnosed according to the 2010 IADPSG criteria during gestational week 24–28 (10). Hypertension, PE, and ICP were all diagnosed and confirmed based on the physician's diagnosis and extracted by the corresponding International Classification of Diseases, 10th Revision (ICD-10) codes.

### Statistical analysis

**Figure 2:**
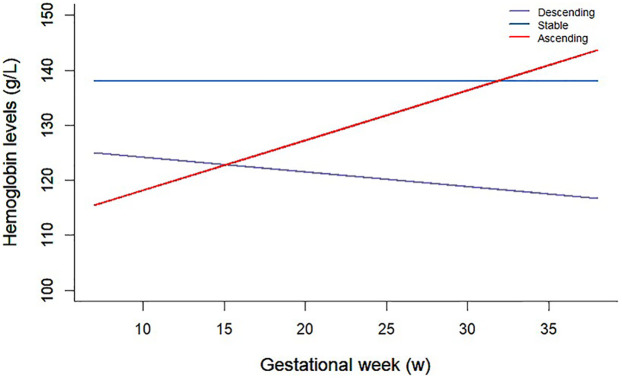
Trajectories of hemoglobin levels during pregnancy and immediate after delivery by latent class analysis.

Continuous variables were assessed for normality using Shapiro–Wilk tests. Normally distributed variables are reported as mean (SD); non-normal variables as median (min-max). Kruskal–Wallis rank-sum test was used to check whether the distribution of variables differs across different hemoglobin trajectory groups. Baseline characteristics were described using means and standard deviations (SD) for continuous variables and numbers and percentages for categorical variables. Baseline characteristics were reported as mean ± SD for continuous variables and as counts (percentage) for categorical variables. Population characteristics were compared across hemoglobin level trajectory groups using one-way ANOVA for continuous variables and Chi-square or Fisher's exact tests for categorical variables. Pairwise comparisons were conducted between the stable and descending groups, and between the stable and ascending groups. Latent class trajectory modeling of hemoglobin levels was conducted using the “lcmm” package in R. with gestational week as the time scale; models with 2–5 classes and linear/quadratic time terms were compared using BIC and interpretability; a random intercept was included; the 3-class solution (stable/descending/ascending) provided the best balance of fit and parsimony (Figure 2). Associations between hemoglobin trajectory groups and binary clinical outcomes were assessed using generalized linear models with a logit link. The clinical outcomes analyzed included PROM, cesarean section, SGA, LGA, preterm birth, preeclampsia, postpartum hemorrhage, and macrosomia. Odds ratios (ORs) and corresponding 95% confidence intervals (CIs) for associations between hemoglobin trajectories, Hb-TITR categories, and adverse pregnancy outcomes were estimated in two models. Model 1 was the crude model. Model 2 adjusted for maternal age, pre-pregnancy BMI, gestational week at delivery and coexisting morbidities during pregnancy (GDM, hypertension, ICP and PE) except for stratified variables in the subgroup analysis. Analyses were further stratified by maternal age and pre-pregnancy BMI to assess potential effect modification. Complete-case analyses were used with no missing values. All statistical analyses were performed using R software (Version 4.3.2), and statistical significance was set at a two-sided *P* value of <0.05.

## Results

### Baseline characteristics

A total of 7,653 participants were included, categorized into three hemoglobin trajectory groups during pregnancy (Table 1): stable (*n* = 6,521), descending (*n* = 798), and ascending (*n* = 334). The mean age of participants was 31.5 ± 3.76 years and was comparable across trajectory groups (*P* = 0.894). However, pre-pregnancy BMI was significantly lower in the descending trajectory group (23.9 ± 6.12 kg/m^2^) compared to stable (28.3 ± 9.10 kg/m^2^) and ascending (28.1 ± 7.32 kg/m^2^) groups (*P* < 0.001). Gestational age at delivery was slightly lower in the descending trajectory group (39.0 ± 1.23 weeks) compared to stable (39.3 ± 0.60 weeks) and ascending groups (39.3 ± 0.77 weeks; *P* < 0.001). The composite adverse pregnancy outcome occurred in 20% of participants, with the highest rate observed in the descending group (49.1%) compared to stable (16.5%) and ascending (19.5%) groups (*P* < 0.001). Individual adverse outcomes including GDM, PE, hypertension, ICP, PROM, C-section, SGA, LGA, preterm birth, preeclampsia, macrosomia, and postpartum hemorrhage differed significantly among the trajectory groups (all *P* < 0.05).

**Table 1:** Baseline characteristics of the study population by different trajectories of hemoglobin during pregnancy.

	Total	Hemoglobin level trajectory group			*P* value
		Stable	Descending	Ascending	
No. of participants	7,653	6,521	798	334	
Age, years	31.5 (3.76)	31.5 (3.75)	31.4 (3.77)	31.6 (3.85)	0.894
Pre-pregnancy BMI, kg/m^2^	27.8 (8.87)	28.3 (9.10)	23.9 (6.12)	28.1 (7.32)	<0.001
Gestational week, w	39.4 (34.0, 41.2)	39.0 (34.2, 41.0)	39.1 (34.0, 41.2)	39.3 (34.1, 41.0)	<0.001
Composite outcome, *n* (%)	1,530 (20%)	107 (16.5%)	392 (49.1%)	65 (19.5%)	<0.001
GDM, *n* (%)	330 (4.3%)	242 (3.7%)	75 (9.4%)	13 (3.9%)	<0.001
PE, *n* (%)	108 (1.4%)	78 (1.2%)	26 (3.3%)	4 (1.2%)	<0.001
Hypertension, *n* (%)	97 (1.3%)	71 (1.1%)	22 (2.8%)	4 (1.2%)	0.001
ICP, *n* (%)	72 (0.9%)	52 (0.8%)	18 (2.3%)	2 (0.6%)	0.001
PROM, *n* (%)	376 (4.9%)	261 (4%)	96 (12%)	19 (5.7%)	<0.001
C-section, *n* (%)	1,052 (13.7%)	730 (11.2%)	272 (34.1%)	50 (15%)	<0.001
SGA, *n* (%)	84 (1.1%)	61 (0.9%)	22 (2.8%)	1 (0.3%)	<0.001
LGA, *n* (%)	127 (1.7%)	88 (1.3%)	34 (4.3%)	5 (1.5%)	<0.001
Preterm, *n* (%)	94 (1.2%)	64 (1%)	24 (3%)	6 (1.8%)	<0.001
Preeclampsia, *n* (%)	51 (0.7%)	37 (0.6%)	11 (1.4%)	3 (0.9%)	0.027
Macrosomia, *n* (%)	127 (1.7%)	87 (1.3%)	34 (4.3%)	6 (1.8%)	<0.001
Postpartum hemorrhage, mL	204 (125, 1,600)	125 (125, 1,100)	300 (300, 1,500)	350 (125, 1,600)	<0.001

Continuous variables were presented as mean (SD) or median (min, max). Categorical variables were presented as *n* (%). GDM, gestational diabetes mellitus; PE, pulmonary embolism; ICP, intrahepatic cholestasis of pregnancy; PROM, premature rupture of membranes; SGA, small for gestational age; LGA, large for gestational age.

### Association between hemoglobin trajectories and adverse outcomes

Compared with participants in the stable hemoglobin trajectory group (Table 2), those in the descending trajectory group had significantly higher odds of experiencing adverse pregnancy outcomes (crude OR 4.90, 95% CI: 4.20–5.72; adjusted OR 3.92, 95% CI: 3.30–4.66, *P* < 0.001). In contrast, participants in the ascending trajectory group did not exhibit significantly elevated risks compared to the stable group (adjusted OR 1.13, 95% CI: 0.83–1.51, *P* = 0.42). Similar associations were observed after stratification by maternal age and pre-pregnancy BMI. In particular, the descending trajectory consistently demonstrated strong associations with adverse outcomes across all subgroups (all *P* < 0.001), whereas the ascending trajectory showed no significant associations.

**Table 2:** Odds ratios of adverse pregnancy outcomes by different trajectories of hemoglobin during pregnancy.

Trajectory	No. of participants	No. of cases	Odds ratios (95% confidence intervals)			
			Model 1	*P* value	Model 2	*P* value
Total population						
Stable	6,521	1,073	1.00		1.00	
Descending	798	392	4.90 (4.20, 5.72)	<0.001	3.92 (3,30, 4.66)	<0.001
Ascending	334	65	1.23 (0.92, 1.61)	0.15	1.13 (0.83, 1.51)	0.42
Age ≥30 years						
Stable	4,519	790	1.00		1.00	
Descending	544	284	5.16 (4.29, 6.21)	<0.001	3.91 (3.17, 4.83)	<0.001
Ascending	235	50	1.28 (0.92, 1.75)	0.14	1.10 (0.76, 1.55)	0.593
Age <30 years						
Stable	2,002	283	1.00		1.00	
Descending	254	108	4.49 (3.40, 5.93)	<0.001	3.67 (2.72, 4.95)	<0.001
Ascending	99	15	1.08 (0.59, 1.85)	0.777	1.12 (0.61, 1.93)	0.687
Pre-pregnancy BMI ≥24 kg/m^2^						
Stable	4,709	717	1.00		1.00	
Descending	293	116	3.65 (2.84, 4.67)	<0.001	2.99 (2.27, 3.93)	<0.001
Ascending	238	44	1.26 (0.89, 1.75)	0.175	1.07 (0.73, 1.53)	0.735
Pre-pregnancy BMI < 24 kg/m^2^						
Stable	1,812	356	1.00		1.00	
Descending	505	276	4.93 (4.00, 6.09)	<0.001	4.59 (3.66, 5.75)	<0.001
Ascending	96	21	1.15 (0.68, 1.85)	0.59	1.18 (0.69, 1.94)	0.522

Model 1: Crude Model. Model 2: Adjusted for maternal age, pre-pregnancy BMI, gestational week at delivery and coexisting morbidities during pregnancy (GDM, hypertension, ICP and PE) except for stratified variables in the subgroup analysis.

### Association between time in target hemoglobin range (Hb-TITR) and adverse outcomes

Participants with less time spent in the target hemoglobin range (Hb-TITR) during pregnancy had higher risks of adverse outcomes (Table 3). Specifically, compared to those maintaining 100% time in target range, participants with Hb-TITR between >80% to <100% had higher adjusted odds of adverse outcomes (OR 1.37, 95% CI: 1.15–1.62, *P* < 0.001), and this risk was even higher for participants with Hb-TITR <80% (adjusted OR 1.35, 95% CI: 1.10–1.65, *P* = 0.004). Analyzed as a continuous variable, each incremental increase in time spent within the target range was associated with lower odds of adverse outcomes (adjusted OR 0.37, 95% CI: 0.23–0.60, *P* < 0.001). Similar associations were observed in analyses stratified by maternal age and pre-pregnancy BMI.

**Table 3:** Odds ratios of adverse pregnancy outcomes by different categories of time in target hemoglobin range (Hb-TITR) during pregnancy.

Hb-TITR, %	No. of participants	No. of cases	Odds ratios (95% confidence intervals)			
			Model 1	*P* value	Model 2	*P* value
Total population						
100	6,047	1,132	1.00		1.00	
>80 to <100	943	227	1.38 (1.17, 1.62)	<0.001	1.37 (1.15, 1.62)	<0.001
<80	663	171	1.51 (1.25, 1.81)	<0.001	1.35 (1.10, 1.65)	0.004
As continuous variable	7,653	1,530	0.29 (0.19, 0.45)	<0.001	0.37 (0.23, 0.60)	<0.001
Age ≥30 years						
100	4,201	841	1.00		1.00	
>80 to <100	634	156	1.30 (1.07, 1.58)	0.008	1.28 (1.03, 1.58)	0.025
<80	463	127	1.51 (1.21, 1.87)	<0.001	1.35 (1.05, 1.71)	0.016
As continuous variable	5,298	1,124	0.26 (0.15, 0.44)	<0.001	0.33 (0.18, 0.59)	<0.001
Age <30 years						
100	1,846	291	1.00		1.00	
>80 to <100	309	71	1.59 (1.18, 2.13)	0.002	1.51 (1.11, 2.04)	0.007
<80	200	44	1.51 (1.04, 2.14)	0.024	1.34 (0.91, 1.93)	0.124
As continuous variable	2,355	406	0.37 (0.17, 0.84)	0.015	0.49 (0.21, 1.16)	0.095
Pre-pregnancy BMI ≥24 kg/m^2^						
100	4,223	667	1.00		1.00	
>80 to <100	611	116	1.25 (1.00, 1.55)	0.046	1.18 (0.93, 1.48)	0.169
<80	406	80	1.31 (1.00, 1.69)	0.041	1.20 (0.91, 1.58)	0.188
As continuous variable	5,240	863	0.35 (0.19, 0.66)	<0.001	0.45 (0.24, 0.89)	0.018
Pre-pregnancy BMI <24 kg/m^2^						
100	1,824	465	1.00		1.00	
>80 to <100	332	111	1.47 (1.14, 1.88)	0.003	1.59 (1.21, 2.06)	<0.001
<80	257	91	1.60 (1.21, 2.10)	<0.001	1.52 (1.12, 2.04)	0.006
As continuous variable	2,413	667	0.31 (0.16, 0.60)	<0.001	0.33 (0.16, 0.67)	0.002

Model 1: Crude Model. Model 2: Adjusted for maternal age, pre-pregnancy BMI, gestational week at delivery and coexisting morbidities during pregnancy (GDM, hypertension, ICP and PE) except for stratified variables in the subgroup analysis.

### Associations with individual adverse outcomes

When evaluating individual adverse pregnancy outcomes (Table 4), participants in the descending hemoglobin trajectory group had significantly higher odds of PROM (OR 2.38, 95% CI: 1.80–3.10), C-section (OR 3.14, 95% CI: 2.60–3.77), SGA (OR 2.63, 95% CI: 1.53–4.36), LGA (OR 2.72, 95% CI: 1.75–4.14), postpartum hemorrhage (OR 2.31, 95% CI: 1.65–3.21), and macrosomia (OR 3.38, 95% CI: 2.19–5.11), compared to the stable trajectory group. There were no significant associations between ascending trajectory and any specific individual adverse outcomes evaluated (all *P* > 0.05).

**Table 4:** Odds ratios of individual adverse pregnancy outcomes by different trajectories of hemoglobin during pregnancy.

Individual components	Adjusted odds ratios (95% confidence intervals)		
	Stable	Descending	Ascending
PROM	1.00	2.38 (1.80, 3.10)	1.35 (0.80, 2.15)
C-section	1.00	3.14 (2.60, 3.77)	1.29 (0.92, 1.77)
SGA	1.00	2.63 (1.53, 4.36)	0.32 (0.02, 1.43)
LGA	1.00	2.72 (1.75, 4.14)	1.07 (0.37, 2.40)
Preterm	1.00	0.71 (0.35, 1.38)	1.15 (0.31,3.33)
Preeclampsia	1.00	1.57 (0.73, 3.13)	1.58 (0.38, 4.42)
Excessive postpartum hemorrhage	1.00	2.31 (1.65, 3.21)	1.35 (0.70, 2.36)
macrosomia	1.00	3.38 (2.19, 5.11)	1.39 (0.54, 2.94)

Adjusted for maternal age, pre-pregnancy BMI, gestational week at delivery and coexisting morbidities during pregnancy (GDM, hypertension, ICP and PE) except for stratified variables in the subgroup analysis.

## Discussion

In this large retrospective study using real-world pregnancy records from a large obstetrics and gynecology specialty clinic in Shanghai, China, we found significant associations between hemoglobin trajectories during pregnancy and the risk of adverse pregnancy outcomes. Women who experienced descending hemoglobin trajectories had substantially higher risks of composite adverse outcomes compared to those with stable hemoglobin levels, whereas ascending hemoglobin trajectories did not significantly increase these risks. Furthermore, we observed an inverse relationship between the proportion of pregnancy duration spent within the target hemoglobin range (Hb-TITR) and the risk of adverse outcomes, underscoring the clinical relevance of maintaining hemoglobin within recommended limits throughout pregnancy.

Our findings indicated that nearly half (49.1%) of women with descending hemoglobin trajectories experienced adverse outcomes, a substantially higher proportion than women with stable (16.5%) or ascending (19.5%) trajectories. These observations align with prior literature highlighting the detrimental impact of significant hemoglobin decline, indicative of maternal anemia, on pregnancy outcomes, possibly due to impaired uteroplacental perfusion, decreased oxygen transport capacity, and compromised uterine muscle function (11–14).

Interestingly, the ascending hemoglobin trajectory group, characterized by gradually increasing hemoglobin levels throughout pregnancy, did not exhibit significantly elevated risks for adverse pregnancy outcomes. Previous studies have suggested potential risks associated with elevated hemoglobin levels, including preeclampsia or fetal growth restriction (15, 16); however, our findings suggested that moderate increases within clinically acceptable ranges might not significantly impact overall risk. Nonetheless, given the relatively small sample size of participants in this trajectory group, these results should be interpreted cautiously. Further investigation with larger cohorts is warranted to clarify potential risks associated with elevated hemoglobin trajectories. Our findings also supported a U-shaped relationship between hemoglobin dynamics and fetal growth, wherein excessive declines in hemoglobin, consistent with anemia or hemodilution beyond physiological adaptation, may compromise placental oxygen delivery and increase the risk of SGA birth, while co-existing metabolic conditions within the same trajectory class may predispose to LGA and macrosomia. This apparent bidirectional risk within the descending-trajectory group likely reflects underlying heterogeneity in maternal phenotypes and pathophysiology rather than a single causal pathway. Taken together, these results indicated that suboptimal hemoglobin control across gestation can be associated with elevated risks at both tails of the fetal growth distribution, underscoring the need for phenotype-specific stratification and targeted intervention trials to clarify mechanisms and optimize perinatal outcomes.

Our study further highlighted the significance of sustained optimal hemoglobin management, as reflected by Hb-TITR. Women who maintained target hemoglobin ranges for less than 80% of pregnancy had significantly higher risks of adverse outcomes compared to those consistently within target ranges, supporting recommendations for regular hemoglobin monitoring and timely interventions during prenatal care. This novel measure of Hb-TITR may thus serve as a useful clinical tool for evaluating and managing anemia risk throughout pregnancy. In subgroup analyses stratified by maternal age and pre-pregnancy BMI, associations remained robust, particularly among older women (≥30 years) and those with higher BMI, reinforcing the importance of personalized hemoglobin management strategies within these potentially vulnerable populations (17, 18).

The association we observed could be understood through several physiological mechanisms. Pregnancy involves complex cardiovascular and hematologic adaptations aimed at supporting fetal development, notably marked by increased plasma volume leading to physiological hemodilution. When hemoglobin levels decline excessively, indicative of maternal anemia, uteroplacental perfusion can become compromised. This impaired perfusion reduces oxygen and nutrient delivery to the developing fetus, increasing the risk of fetal hypoxia, growth restriction, and preterm birth (19, 20). Additionally, maternal anemia may weaken uterine muscle function, thereby elevating the risk for postpartum hemorrhage and preterm rupture of membranes (21). Conversely, elevated hemoglobin concentrations can signal inadequate plasma volume expansion or hemoconcentration. Such elevated levels may increase maternal blood viscosity and vascular resistance, impairing placental blood flow and limiting oxygen transport (22, 23). This pathophysiological condition has been linked to heightened risks of preeclampsia and fetal growth restriction due to compromised uteroplacental exchange efficiency. Thus, maintaining stable hemoglobin levels within clinically recommended ranges is essential to ensure optimal maternal cardiovascular adaptation, placental perfusion, and oxygen delivery, thereby minimizing risks of adverse maternal and neonatal outcomes (24). Our findings reinforce the importance of closely monitoring hemoglobin dynamics throughout pregnancy to inform timely clinical interventions aimed at mitigating these physiological risks.

The strengths of our study included a large sample size and comprehensive clinical data collection from a specialized obstetric population. Additionally, we introduced Hb-TITR, a time-weighted measure of the proportion of gestation spent within trimester-specific hemoglobin targets, moving beyond single-time-point values to capture exposure intensity and duration. This improves physiological fidelity and clinical interpretability. However, several limitations should be considered. Firstly, the retrospective design inherently introduced potential biases related to data accuracy and completeness. Secondly, our study population was derived from a single urban OBGYN clinic in Shanghai, limiting generalizability to broader populations and different geographic areas. Lastly, despite adjusting for key confounders, residual confounding by unmeasured factors such as nutritional status, adherence to iron supplementation, and underlying chronic health conditions could not be completely ruled out.

In conclusion, our study demonstrated that a descending hemoglobin trajectory during pregnancy was significantly associated with increased risk of adverse pregnancy outcomes, whereas maintaining stable hemoglobin levels substantially mitigated these risks. These results underscore the clinical value of continuous hemoglobin monitoring and support the integration of trajectory analysis and Hb-TITR assessment into routine prenatal care practice. Further prospective studies are necessary to validate these findings and establish optimal hemoglobin management protocols across diverse populations.

## Data Availability

The raw data supporting the conclusions of this article will be made available by the authors, without undue reservation.
